# Supplementary material for: A network dynamical simulation model for the study of antibiotic resistance in microbial communities
Source: Gut Microbes. 2026 Sep 19;18(1):2734703. doi: 10.1080/19490976.2026.2734703 (PMC13596919; doi:10.1080/19490976.2026.2734703)
Supplement: Supplementary file 1 [file KGMI_A_2734703_SM9053.zip › supplementary_file_1/Figures/er_pct_delta_n_edges_n300.pdf]

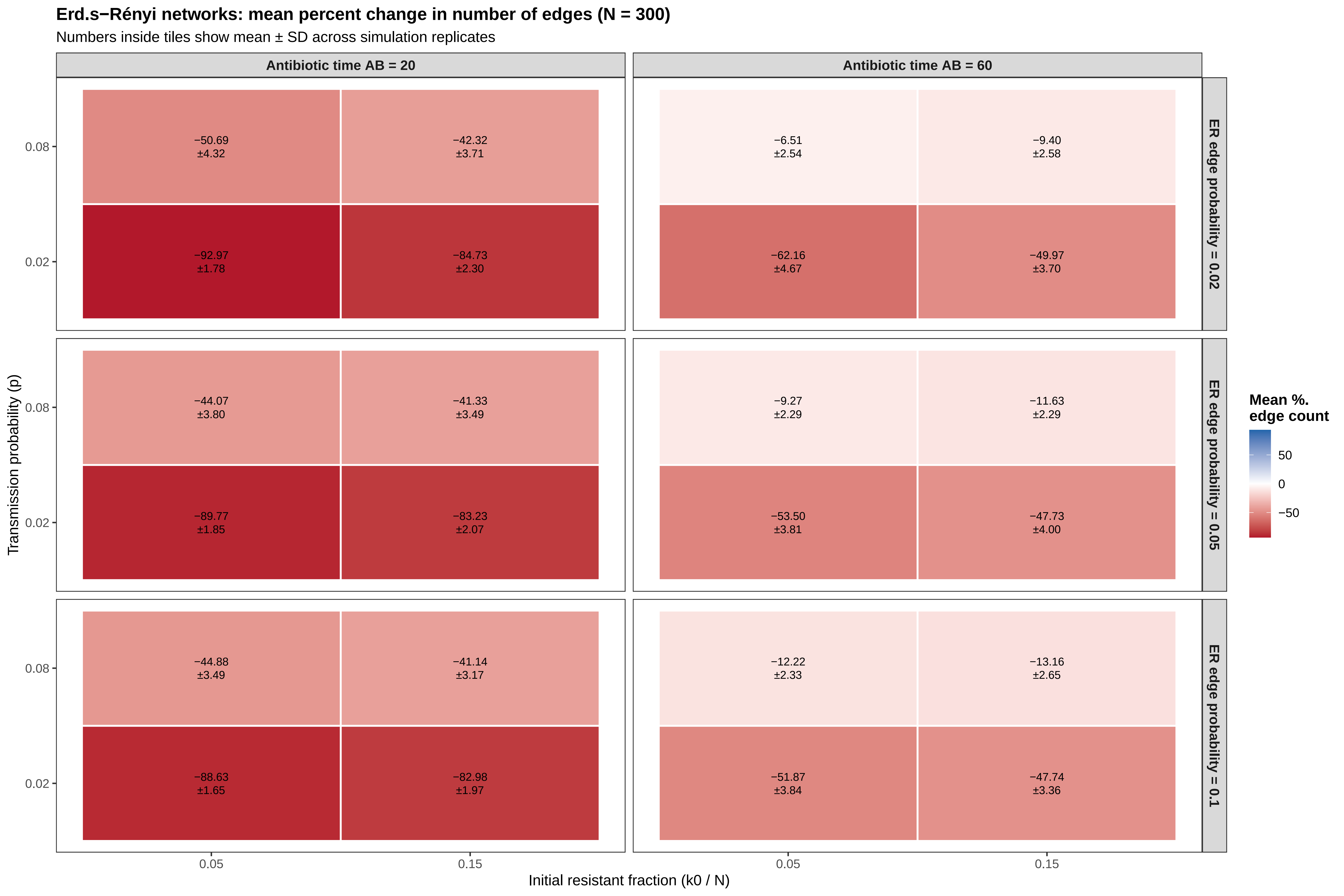

Erd.s−Rényi networks: mean percent change in number of edges (N = 300)
Numbers inside tiles show mean ± SD across simulation replicates
Antibiotic time AB = 20
Antibiotic time AB = 60
ER edge probability = 0.02
ER edge probability = 0.05
ER edge probability = 0.1
Transmission probability (p)
0.08
0.02
Initial resistant fraction (k0 / N)
0.05
0.15
−50.69 ±4.32
−42.32 ±3.71
−92.97 ±1.78
−84.73 ±2.30
−6.51 ±2.54
−9.40 ±2.58
−62.16 ±4.67
−49.97 ±3.70
−44.07 ±3.80
−41.33 ±3.49
−89.77 ±1.85
−83.23 ±2.07
−9.27 ±2.29
−11.63 ±2.29
−53.50 ±3.81
−47.73 ±4.00
−44.88 ±3.49
−41.14 ±3.17
−88.63 ±1.65
−82.98 ±1.97
−12.22 ±2.33
−13.16 ±2.65
−51.87 ±3.84
−47.74 ±3.36
Mean %. edge count
50
0
−50
